# Effect of Prandtl Number on Mixed Convective Heat Transfer from a Porous Cylinder in the Steady Flow Regime

**DOI:** 10.3390/e22020184

**Published:** 2020-02-06

**Authors:** Shimin Yu, Tingting Tang, Jianhui Li, Peng Yu

**Affiliations:** 1 Harbin Institute of Technology, Harbin 150001, China; 11849524@mail.sustech.edu.cn; 2 Shenzhen Key Laboratory of Complex Aerospace Flows, Department of Mechanics and Aerospace Engineering, Southern University of Science and Technology, Shenzhen 518055, China; tangtt@sustech.edu.cn (T.T.); lijh@sustech.edu.cn (J.L.); 3 Center for Complex Flows and Soft Matter Research, Southern University of Science and Technology, Shenzhen 518055, China; 4 Guangdong Provincial Key Laboratory of Turbulence Research and Applications, Department of Mechanics and Aerospace Engineering, Southern University of Science and Technology, Shenzhen 518055, China

**Keywords:** porous cylinder, mixed convection, the Prandtl number, the recirculating wake, heat transfer

## Abstract

The effect of the Prandtl number (*Pr*) on the flow and heat transfer from a porous circular cylinder with internal heat generation in the mixed convection regime is numerically investigated. The steady flow regime is considered over the ranges of the Reynolds number (*Re*), Darcy number (*Da*), and Richardson number (*Ri*), varying from 5 to 40, 10^−6^ to 10^−2^, and 0 to 2, respectively. The wake structure, the temperature distribution, and the heat transfer rate are discussed. Besides precipitating the growth of the recirculating wake, the Prandtl number is found to have a significant impact on the thermal characteristics. The concave isotherms, resembling a saddle-shaped structure, occur behind the cylinder at larger *Pr*, resulting in swells of the isotherms pairing off at the lateral sides. These swells are found to have a negative effect on heat transfer owing to a relatively smaller temperature gradient there. Then, the heat transfer rate in terms of the local Nusselt number (*Nu*) and enhancement ratio (*Er*) is calculated, which is closely related to *Pr*, *Re*, *Da*, and *Ri.* The local minimum heat transfer rate along the cylinder surface is found at the position where the swells of the isotherms form.

## 1. Introduction

The flow pattern and heat transfer characteristics around and through a porous cylinder immersed in a free stream have attained great academic significance and attracted extensive attention owing to the wide engineering applications [1]. Such transport processes commonly appear in heat exchangers, cooling and heating for food, nuclear biological chemical equipment, catalytic chemical reactors, metal melting and solidification, flow through and around microchannels, as well as electronic components [2,3,4]. Porous material exhibits its potential for the augmentation in the heat transfer rate due to the high effective thermal conductivity [5] and its specific structure of increasing the heat transfer area between the solid and fluid regions [6,7]. Dhinakaran and Ponmozhi [8] found that compared to that for a solid cylinder, a considerable heat transfer increment was achieved for a permeable cylinder, since more fluid passed through the porous cylinder, carrying more heat away. Besides, a porous medium provides an ability of controlling the flow characteristics and suppressing the formation of vortex shedding [9]. For example, Al-Sumaily and Thompson [10] found that for pulsatile flow over a circular cylinder in a channel with the presence of the porous media, a highly stable flow occurs without the formation of the extended wake in the flow field. The effect of porous media on the heat transfer augmentation from the cylinder is more significant than that promoted by the pulsating flow [10].

Heating can inherently control the evolution of the boundary layer around a bluff body. When fluid flows around a heated obstacle, the thermal buoyancy is generated, which significantly influences the formation and the separation of the momentum boundary layer as well as the heat transfer rate around the bluff obstacle [11]. As far as the thermal buoyancy is concerned, previous works are mostly focused on the flow and heat transfer from a bluff body in the mixed convection regime [12,13,14,15,16]. However, most of them were concentrated on a solid counterpart, and relatively few investigations were carried out for a porous counterpart. For instance, Wu and Wang [17] numerically analyzed the mixed convective heat transfer from a heated porous cylinder in the laminar flow for different channel-to-cylinder height ratios at the Reynolds number (*Re*) of 250. Their results showed that the heat transfer rate increases with an increase in the strength of buoyancy, and the buoyancy effect on the local heat transfer is clearer for the lower channel-to-cylinder height ratio than for the higher ratio. More recently, Yu et al. [18] investigated the effect of thermal buoyancy on the mixed convective flow and heat transfer through and around a porous circular cylinder with internal heat generation. They revealed that the thermal buoyancy has two opposite effects on the wake structure and heat transfer performance, which is significantly dependent on the interaction between the incoming flow and buoyancy directions.

Moreover, additional attempts can be made to further increase the heat transfer rate by applying different working fluids with various thermal conductivities, i.e., considering the effect of Prandtl number (*Pr*). Dhiman et al. [19] carried out the numerical simulations for both constant surface temperature and constant heat flux cases and studied the effect of *Pr* (0.7 ≤ *Pr* ≤ 4000) on the flow and heat transfer from a heated solid cylinder in the steady flow regime (1 ≤ *Re* ≤ 45). Their results showed that the average Nusselt number (*Nu_ave_*) around the cylinder surface increases monotonically with an increase in *Re* and/or *Pr* and is higher in the constant heat flux case than that in the other case. Ajith Kumar et al. [20] found that the vortices in the recirculating wake behind a solid circular cylinder disappear beyond a critical *Pr*, but they reappear over another critical *Pr* for a fixed *Re* and *Ri* (the Richardson number) in the mixed convection regime. Besides, the thermal boundary layer thickness decreases with increasing *Pr*. Other previous works about the effect of *Pr* are available by Dennis et al. [21], Vilimpod et al. [22], Bharti et al. [23], Dhiman et al. [24], Sahu et al. [25], Sharma et al. [26], Chen et al. [27], and Sufyan et al. [28], etc.

Evidently, the above works in the literature indicate that the effect of *Pr* on the flow and heat transfer has been well investigated for the solid counterpart with constant surface temperature or uniform heat flux. However, scant relevant efforts have been made for porous bluff bodies despite the studies of Kim and Jang [29] on the local thermal non-equilibrium model and Anirudh and Dhinakaran [30] on a porous square cylinder in the forced convection regime. Especially, very few studies about the effect of *Pr* have been conducted for a porous circular cylinder with internal heat generation in the mixed convection regime. Therefore, the purpose of the present study is to investigate the effect of *Pr* on this flow and heat transfer configuration. Moreover, a porous material, different from the solid counterpart, can provide passage for thermal fluid [31] and thus enhance the heat transfer rate [18]. The transport phenomenon in porous media with internal heat generation is found extensively in practical applications, for instance, heat generation in electronic elements [32], nuclear reactors [33], and endothermic and exothermic reactions in chemical processes [34,35]. Thus, our study may provide some useful guidance to improve the performance of related thermal engineering applications.

## 2. Problem Statement and Mathematical Formulation

**Figure 1:**
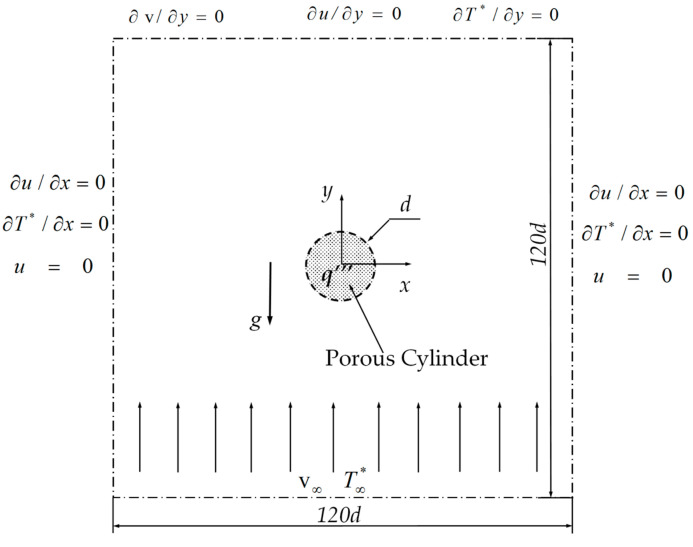
Schematic of flow past a heated porous cylinder.

The present problem with the computational domain is depicted in Figure 1, which shows a circular porous cylinder of diameter *d* exposed to a free stream with uniform velocity $v_{\infty }$ and constant temperature $T_{\infty }^{*}$. The velocity direction is consistent with the *y* direction, which is opposite to that of the gravity (*g*). This cylinder with heat generation (*q*′″) is placed at the center of the fictitious domain, whose center coincides with the origin of the coordinates (0, 0). The computational domain is a fixed square with a size 120 times the diameter of the cylinder, which is sufficiently large to reduce the effects of the inlet, outlet, and two lateral boundary conditions.

The governing equations, boundary conditions, and numerical results in the present study are presented in the non-dimensional forms. The diameter of cylinder (*d*), the incoming flow velocity $v_{\infty }$, and $\rho v_{\infty }^{2}$ are used as the length, velocity, and pressure scales, respectively. The non-dimensional temperature is based on both the temperature difference and the magnitude of heat generation. Therefore, the non-dimensional variables are defined as

$$X=\frac{x}{d},\;Y=\frac{y}{d},\;U=\frac{u}{v_{\infty }},\;V=\frac{v}{v_{\infty }},\;P=\frac{p}{\rho v_{\infty }^{2}},\;T=\frac{T^{*}-T^{*}{}_{\infty }}{q^{\prime \prime \prime }d^{2}/k_{f}},$$

where *x* and *y* are the dimensional length in the horizontal and vertical directions, respectively, in Cartesian coordinates; *u* and v are the dimensional velocities along the *x* and *y* directions; *p* and T* are the pressure and temperature, respectively; *k_f_* is the thermal conductivity of fluid; *X*, *Y*, *U*, *V*, *P*, and *T* are the corresponding non-dimensional variables.

The Reynolds number is defined as

(1) $$Re=\frac{v_{\infty }d}{\upsilon },$$

where *υ* is the fluid kinematic viscosity.

The Darcy number (*Da*) is based on the permeability and the diameter of the porous cylinder, which is defined as

(2) $$Da=\frac{K}{d^{2}},$$

where *K* denotes the permeability of the porous cylinder.

In the thermal convection problem, the Richardson number is defined as

(3) $$Ri=\frac{Gr}{{Re}^{2}},$$

where *Gr* is the Grashof number, which is expressed as *Gr* = *gβq*′″*d*^5^/*k_f_υ*^2^ for the heat-generation case. Here, *β* is the coefficient of thermal expansion.

Considering the two-dimensional, steady, and incompressible flow, the non-dimensional governing equations with the Boussinesq approximation and negligible dissipation can be expressed as the continuity equation

(4) $$\frac{\partial U}{\partial X}+\frac{\partial V}{\partial Y}=0,$$

the momentum equations

(5) $$\begin{array}{ll} \frac{1}{\varepsilon^{2\phi }}\left(U\frac{\partial U}{\partial X}+V\frac{\partial U}{\partial Y}\right) & =-\frac{\partial P}{\partial X}+\frac{1}{\varepsilon^{\phi }Re}\left(\frac{\partial^{2}U}{\partial X^{2}}+\frac{\partial^{2}U}{\partial Y^{2}}\right)\\ & -\phi \frac{U}{Re\cdot Da}-\phi \frac{1.75}{\sqrt{150}}\frac{\sqrt{U^{2}+V^{2}}}{\sqrt{Da}}\frac{U}{\varepsilon^{3/2}} \end{array}$$

(6) $$\begin{array}{ll} \frac{1}{\varepsilon^{2\phi }}\left(U\frac{\partial V}{\partial X}+V\frac{\partial V}{\partial Y}\right) & =-\frac{\partial P}{\partial Y}+\frac{1}{\varepsilon^{\phi }Re}\left(\frac{\partial^{2}V}{\partial X^{2}}+\frac{\partial^{2}V}{\partial Y^{2}}\right)\\ & -\phi \frac{V}{Re\cdot Da}-\phi \frac{1.75}{\sqrt{150}}\frac{\sqrt{U^{2}+V^{2}}}{\sqrt{Da}}\frac{V}{\varepsilon^{3/2}}\\ & +Ri\cdot T \end{array}$$

and the energy equation

(7) $$U\frac{\partial T}{\partial X}+V\frac{\partial T}{\partial Y}=\frac{R_{c}{}^{\phi }}{Re\cdot Pr}\left(\frac{\partial^{2}T}{\partial X^{2}}+\frac{\partial^{2}T}{\partial Y^{2}}\right)+\frac{\phi }{Re\cdot Pr},$$

where *ε* is the porosity of the porous cylinder; *Pr* is the Prandtl number, defined as υ/α, where α is the thermal diffusivity; *R_c_* is the ratio of thermal conductivity in the fluid saturated porous region, which is defined as *k_e_*/*k_f_* (*k_e_* is the effective thermal conductivity of fluid-saturated porous cylinder); ϕ is a binary constant, which is expressed as

$$\phi =\{\begin{array}{c} 0,\;\mathrm{for}\;\mathrm{fluid}\;\mathrm{region}\\ 1,\;\mathrm{for}\;\mathrm{porous}\;\mathrm{region} \end{array}$$

The relevant boundary conditions used in the present study are presented in Figure 1. To obtain the closed forms of the governing equations, the interfacial boundary conditions for flow and heat transfer at the porous–fluid interface are needed. For the flow at the interface, the stress jump condition and continuities of velocity and normal stress are applied [36,37,38]. For heat transfer, the continuities of temperature and heat flux are implemented at the interface between porous and fluid regions. The detailed address for interfacial boundary conditions was provided by the studies of Yu et al. [39] and Chen et al. [40].

## 3. Grid-Independence Analysis and Code Validation

**Figure 2:**
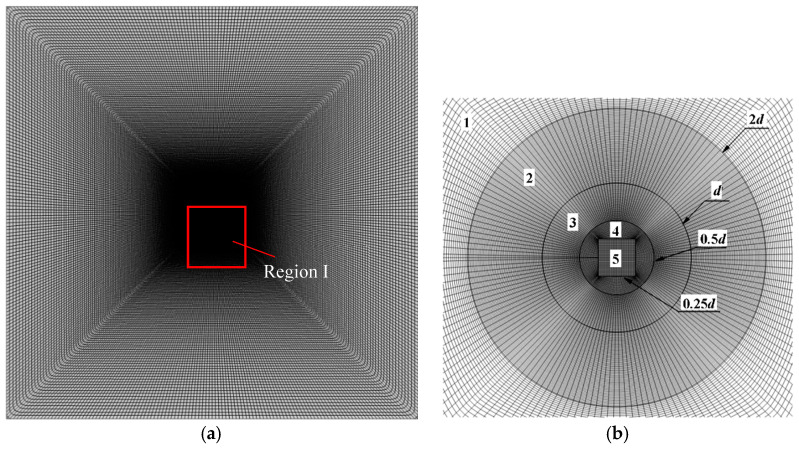
A typical example of the grid: (**a**) the grid for the whole computational domain; (**b**) a closer view of Region I with mesh inside and around the porous cylinder and the domain partitioning.

In the present study, a C++ code, originally developed by Yu et al. [39], based on the finite volume method with the collocated body-fitted and multi-block grids is adopted. The numerical simulations are performed in the Linux computing cluster system, which is equipped with total 1630 Intel(R) Xeon(R) Gold 6140 CPU processors (2.40 GHz/20 cores). To generate smooth body-fitted and structured grids around the porous cylinder surface, the whole computational domain is divided into five blocks. Blocks 1 and 2 comprise the pure fluid region, and blocks 3, 4, and 5 represent the porous regions. Blocks 2, 3, and 4 are meshed by using an O-grid to guarantee orthogonal grids around the cylinder surface with good quality. The grid for the whole computational domain is shown in Figure 2a. A closer view of Region I with mesh inside and around the porous cylinder and the domain partitioning is provided in Figure 2b.

The four sets of cases with different grid configurations presented in Table 1 are considered to conduct the grid-independence study. Numerical results of *Nu_ave_* of such different grid configurations for *Re* = 40, *Da* = 10^−4^, *Ri* = 1, and *Pr* = 1 are also shown in Table 1. The grid convergence index (GCI) [41] is calculated to evaluate the grid convergence, which is expressed as

(8) $$GCI=F_{s}\left|E\right|,$$

where *F_s_* is a safety factor and is estimated to be 1.25 for three or more grid solutions [42,43]. *E* is a fine grid Richardson error estimator, which is defined as

(9) $$E=(f_{2}-f_{1})/(1-r^{p}),$$

where *f*_2_ is a coarse grid solution with grid spacing *h*_2_, and *f*_1_ is a fine grid solution with grid spacing *h*_1_. The refinement factor *r* is calculated from *h*_2_/*h*_1_. *p* presents the formal order of accuracy of the algorithm. In this study, *h*_1_ and *h*_2_ are obtained from the grid spacing on the porous cylinder surface, and *p* is approximately two, owing to a second-order method [41].

Table 1 shows that the GCI (in round brackets) is less than 1% for all mesh configurations, which indicates that these four sets of grid configurations are fine for the present study. The GCI calculated from the third and fourth sets of grids is slightly larger than that from the second and third sets of grids, which may be due to the additional errors from the calculation and integration of the local Nusselt number. To ensure the better resolution and accuracy, the grid size of Case 4 is chosen for the final simulations.

**Figure 3:**
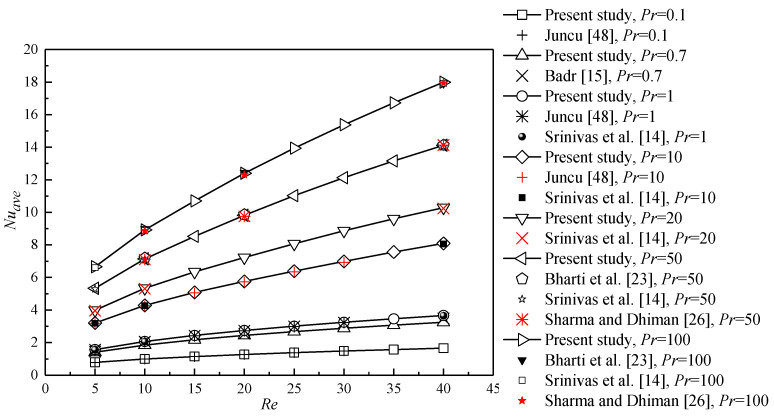
Comparison of the present results with the previous results in the literature.

The numerical model and method used in the present study have been successfully applied for the studies of flow around and through bluff porous bodies with various shapes [44,45,46,47] and heat transfer from a porous circular cylinder [18]. To further investigate the validity of the present numerical method, the computational simulations are performed for a heated solid cylinder with a constant surface temperature at different *Pr*. The local Nusselt number is defined as *Nu* = $-\frac{\partial T}{\partial n}$ (*n* is the normal direction from the porous region to the fluid region), which is consistent with that for a solid cylinder with a constant surface temperature in the previous works. Figure 3 shows the *Nu_ave_* of the present study as a function of *Re* at different *Pr*, which is in good agreement with the previous data provided by Juncu [48], Badr [15], Srinivas et al. [14], Bharti et al. [23], and Sharma and Dhiman [26].

## 4. Results and Discussions

Numerical simulations are performed for different *Pr* (varying from 1 to 100 for different working fluids covering gas, water, light organic fluid, and oil) under the effects of *Re*, *Da*, and *Ri*, varying from 5 to 40, 10^−6^ to 10^−2^, and 0 to 2, respectively. The wake structure, temperature distribution, and heat transfer rate influenced by *Pr* in the mixed convection regime are mainly investigated. Note that the contours of the streamlines and isotherms are arranged horizontally for a better layout. The velocity direction is always consistent with the *y* direction while opposite to the gravity direction.

### 4.1. Flow Pattern

**Figure 4:**
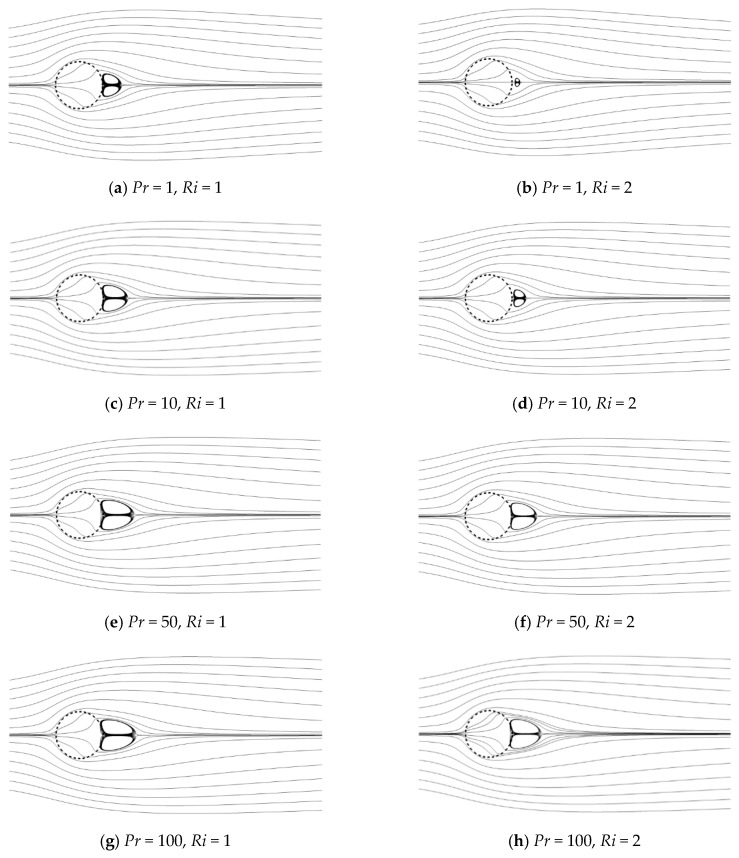
Streamlines for different Prandtl number (*Pr*) values at a Reynolds number (*Re*) = 20 and Darcy number (*Da*) = 10^−3^.

Figure 4 presents the effect of *Pr* on the flow pattern for *Ri* = 1 and 2 at constant *Re* = 20 and *Da* = 10^−3^. The left column presents the streamlines for *Ri* = 1. For this smaller *Ri*, a pair of recirculating wake forms and partially penetrates the porous cylinder from the rear in the present range of *Pr*. When *Pr* varies from 1 to 100, the recirculating wake is elongated along the flow direction and widened in the lateral sides. For a larger *Ri* = 2 shown in the right column, the size of the recirculating wake suffers from a reduction for all *Pr* compared to that for a smaller *Ri*. Especially at a small *Pr*, a pair of very small recirculating wakes occurs and is detached from the rear of the porous cylinder. When *Pr* increases, this recirculating wake moves toward the cylinder and finally also partially penetrates the rear surface at a large *Pr*. To some extent, *Pr* precipitates the growth of the recirculating wake.

**Figure 5:**
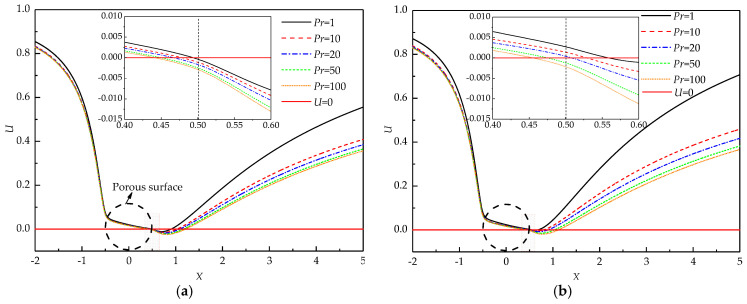
U velocity distribution along the horizontal axis of the cylinder at *Re* = 20 and *Da* = 10^−3^; (**a**) *Ri* = 1, (**b**) *Ri* = 2.

To further investigate the variation of the wake structure with *Pr*, the *U* velocity distribution along the horizontal centerline of the porous cylinder at different *Pr* is depicted in Figure 5. A portion of fluid penetrates the cylinder with nonzero *U* velocity and bleeds from the rear part of the cylinder. The *U* velocity yielding from the surface resembles base bleed [49]. The bleeding from the cylinder partially satisfies the entrainment needs of the shear layer. Thus, the recirculating wake becomes narrower and shorter. The negative velocity is witnessed for all *Pr* at *Re* = 20, *Da* = 10^−3^, and *Ri* = 1 (see Figure 5a), which means the formation of the recirculating wake. The length of the region of the negative *U* velocity along the flow direction represents the whole length of the wake. An increase in *Pr* certainly results in an increment in the wake length. The negative exit velocity is also observed for all *Pr*, which indicates that a part of the recirculating wake penetrates the porous cylinder from the rear surface.

For a large *Ri* = 2 (see Figure 5b), the negative velocity occurs at a distance away from the cylinder for *Pr* ≤ 20, which means the formation of the detached recirculating wake. When *Pr* increases to 50 and 100, the exit velocity becomes negative, indicating that the recirculating wake partially penetrates the porous cylinder. The exit *U* velocity at the rear surface decreases with *Pr*, which indicates a reduction in base bleed. Less fluid is entrained into the near-wake region, which is incapable of supporting the shear flow. As a result, the large recirculating wake should form. *Pr* is defined as the ratio of momentum diffusivity to thermal diffusivity. When *Pr* increases but *Re* is fixed, the thickness of the thermal boundary layer decreases, which enhances the heat transfer rate. Therefore, much heat is carried away from the cylinder, and correspondingly, the temperature of the cylinder decreases. For a large *Pr*, the temperature is low due to the thin thermal boundary layer around the porous cylinder. Correspondingly, the buoyancy term becomes insignificant according to the *Y*-momentum equation, which weakens the effect of thermal buoyancy. Thus, the velocity of fluid flow decelerates. The large recirculating wake forms due to the insufficient entrainment.

**Figure 6:**
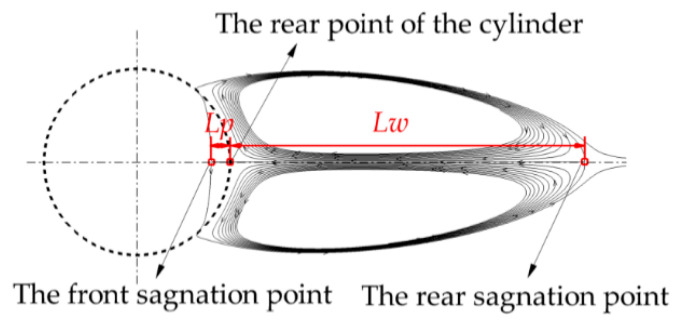
A typical example of the wake structure.

Figure 6 presents a typical structure of a pair of the recirculating wakes. Different from the solid case, the front stagnation point of the recirculating wake may occur inside the porous cylinder. The distance from the front stagnation point of the recirculating wake to the rear point of the cylinder is defined as the penetration depth (*Lp*). The wake length (*Lw*) is measured by the distance from the rear point of the cylinder to the rear stagnation point of the recirculating wake. The entire length of the wake is the summation of *Lw* and *Lp*.

**Figure 7:**
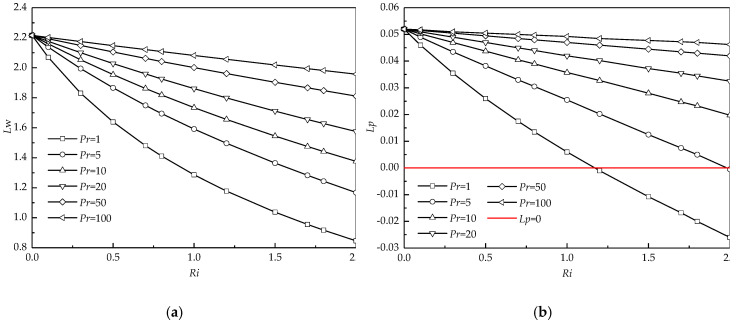
Variations of the wake length (**a**) and the penetration depth (**b**) with Richardson numbers (*Ri*) for different *Pr* at *Re* = 40 and *Da* = 10^−3^.

As stated above, *Pr* has a significant impact on the size of recirculating wake as well as the penetration depth. For this purpose, the detailed comparisons of variations of the wake length (*Lw*) and the penetration depth (*Lp*) with *Ri* at different *Pr* are presented in Figure 7. For all *Ri* (>0), *Lw* obviously increases with *Pr*. This increasing phenomenon becomes significant at large *Ri* (see Figure 7a). For all *Pr*, *Lw* presents a decreasing trend with increasing *Ri*. The variation of *Lw* with *Ri* becomes less sensitive when *Pr* increases, particularly at *Pr* = 100.

Figure 7b presents *Lp* as a function of *Ri* at different *Pr*. The variations of *Lp* with *Pr* and *Ri* are similar to those of *Lw*. It is worth noting that the negative *Lp* occurs for *Pr* ≤ 5 at *Ri* = 2. Negative *Lp* means the formation of the detached recirculating wake, as shown in Figure 4b. When *Pr* varies from 5 to 1, this negative *Lp* increases in magnitude, which indicates that the recirculating wake moves far away from the cylinder with decreasing *Pr*.

### 4.2. Temperature Field

**Figure 8:**
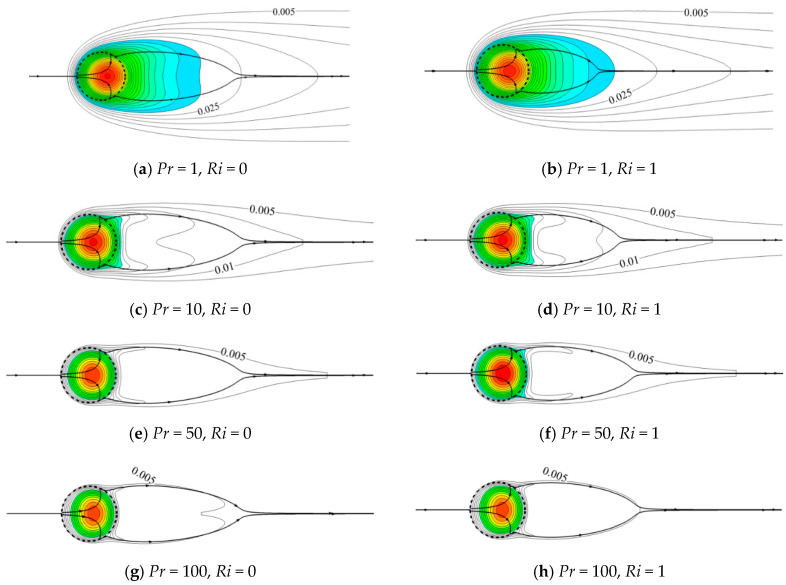
Isotherms (∆*T* = 0.005) for different *Pr* and *Ri* at *Re* = 40 and *Da* = 10^−5^ (the bold lines with arrowheads represent the streamlines).

Since there is a uniform heat source inside the porous cylinder, the compact isotherms distribute around the cylinder surface, especially at the front of the cylinder. Figure 8 shows the representative variations of thermal patterns with *Pr* and *Ri* at constant *Re* = 40 and *Da* = 10^−5^. To better understand the temperature distribution, only the isotherms for *T* ≥ 0.005 are presented. The colored contours are regarded as the thermal plume in our study, which is defined as the high temperature region for *T* ≥ 0.03. The temperature difference between two neighboring isotherms (*∆T*) is 0.005. The bold lines with arrowheads represent the streamlines. The left column illustrates the variations of isotherms with *Pr* in the forced convection regime (*Ri* = 0). Different from flow patterns, isotherms are significantly sensitive to *Pr* at *Ri* = 0. At a small *Pr* (for fixed *Re*), the thermal boundary layer is thick. Correspondingly, the temperature gradient is small, and isotherms sparsely distribute around the cylinder. The heat is also converted downstream by the fluid flow. Therefore, a large thermal plume with sparse isotherms around the cylinder is noticed at *Pr* = 1. When *Pr* increases, the thickness of the thermal boundary layer decreases. Correspondingly, the temperature gradient increases, and thus isotherms tightly assemble in the vicinity of the porous cylinder. An increase in the temperature gradient also results in an increment in the entropy generation, since the entropy generation is proportional to the temperature gradient [50]. Different from the smooth isotherms presented at small *Pr* = 1, the isotherms obviously bifurcate and become concave, resembling a saddle-shaped structure, behind the cylinder at a large *Pr* of 10. In this situation, swells of the isotherms pair off at the lateral sides, which almost align with the streamlines where the flow separation occurs [20]. Besides, a few of the isotherms embrace the cylinder surface, and the area surrounded by the isotherms decreases in size. With a further increase in *Pr*, these phenomena become more obvious. Moreover, at the largest *Pr* = 100, the isotherms start to become concave in shape at around lower *T* = 0.005 compared to other cases of smaller *Pr*, which indicates that the concave isotherms are more likely to occur with increasing *Pr*.

The right column presents the comparison of isotherms at different *Pr* in the mixed convection regime (*Ri* = 1). With the presence of thermal buoyancy, the additional momentum provided by buoyancy compels more fluid to carry away more heat from the cylinder. The isotherms shrink and embrace tightly the cylinder for a fixed *Pr*, which contributes to a larger temperature gradient. The thermal plume also becomes narrow in the lateral direction and evolves in the flow direction. Moreover, a smaller number of isotherms are observed behind the cylinder with increasing *Pr*. The isotherms also become denser with *Pr*, which indicates an increasing temperature gradient.

**Figure 9:**
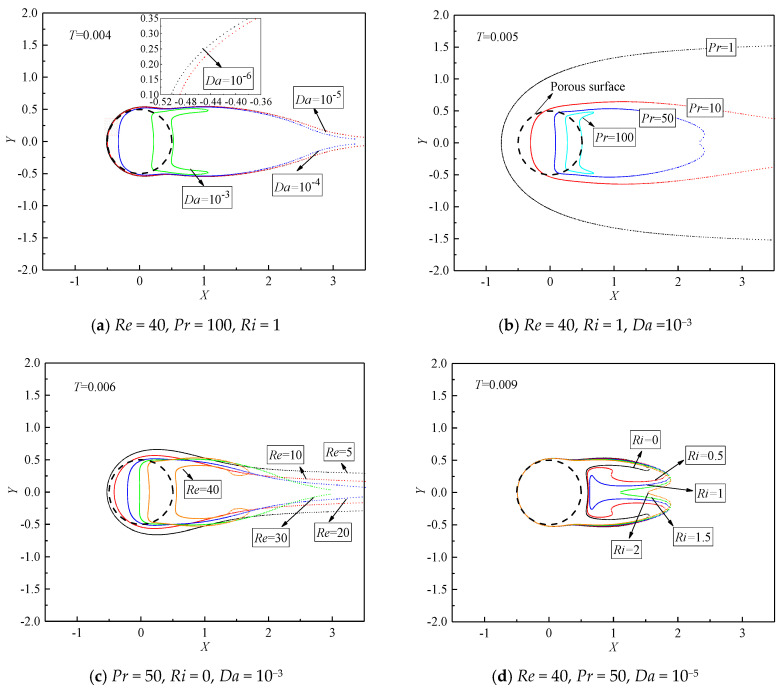
The variations of isothermal structures at different *Pr*, *Re*, *Ri*, and *Da*.

To further investigate the evolutions of the concave isotherms behind the porous cylinder in different conditions, the isotherms of *T* = 0.004, 0.005, 0.006, and 0.009 at various *Da*, *Pr*, *Re*, and *Ri* are illustrated in Figure 9. When the porous cylinder becomes much more permeable, a larger amount of fluid penetrates the porous cylinder and converts much more heat downstream, which is consistent with the cases of Figure 9a. The narrow and contractive isotherm is observed at *Da* = 10^−3^, which indicates a high temperature gradient. With a decrease in *Da*, the isotherm spatially grows in the lateral sides and stretches in the downstream direction. The obvious saddle-shaped isotherm is noticed at *Da* = 10^−3^, which indicates that the isotherms with lower *T* initially start to become concave in shape at larger *Da* compared to the cases of lower *Da*. Indeed, this phenomenon is also valid for other cases of different *Re*, *Pr*, and *Ri*.

Figure 9b shows the variations of the isothermal structure (*T* = 0.005) with *Pr* at constant *Re* = 40, *Ri* = 1, and *Da* = 10^−3^. At the same temperature level, the occurrence of the concave isotherm is witnessed at higher *Pr*. As presented in Figure 9b, the isotherm of *T* = 0.005 becomes concave behind the cylinder for *Pr* ≥ 50. When *Pr* increases, the concave isotherms become significant, and the lateral distribution of the isotherm obviously reduces in width. Similar phenomena are also observed when considering the effect of *Re* on the variation of isotherms, as shown in Figure 9c. The concave structure of the isotherm is highlighted at higher *Re* = 40. A different trend is seen for *Ri* variation (Figure 9d). The thermal buoyancy attempts to relax and soothe the concave structure of the isotherms. At *Ri* = 0, the isotherm of *T* = 0.009 obviously bifurcates and becomes concave. With an increase in *Ri*, these swells of the isotherms tend to expand toward the horizontal centerline and spread in the downstream direction, which interprets the phenomenon for *Ri* varying from 0 to 1 at *Pr* = 50, as shown in Figure 8. Eventually, the concave isotherms behind the cylinder tend to relax at larger *Ri*.

**Figure 10:**
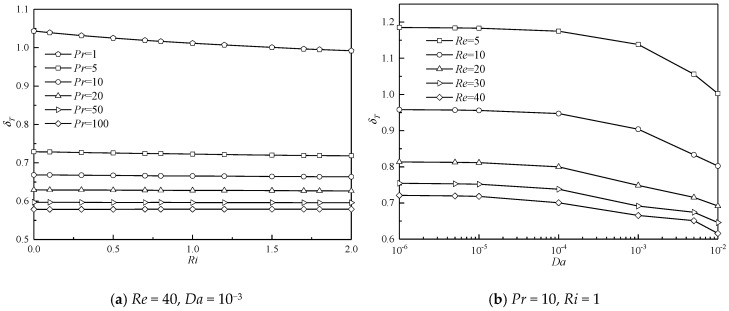
Results of thermal boundary layer at a specific surface point ((*X*, *Y*) = (0, 0.5)).

As stated above, the isotherms spatially expand or shrink in the lateral sides in the influences of various *Da*, *Pr*, *Re*, and *Ri*. Changes of the isotherms in space also result in the variation of the thermal boundary layer. Therefore, the thermal boundary layer thickness is analyzed in the present study. The thickness of the thermal boundary layer (δ_T_) at any location along the surface is defined as the distance from the surface at which the temperature difference T* − $T_{s}^{*}$ = 0.99($T_{\infty }^{*}$ − $T_{s}^{*}$) [51]. Generally, the thermal boundary layer thickness increases in the flow direction for a steady flow around a cylinder. The thermal boundary layer thickness at a specific surface point ((*X*, *Y*) = (0, 0.5)) is monitored. The results are presented in Figure 10.

Figure 10a shows δ_T_ as a function of *Ri* at different *Pr* for the case at constant *Re* = 40 and *Da* = 10^−3^. For all *Pr*, δ_T_ varies monotonously and linearly with *Ri*. For a small *Pr*, δ_T_ obviously experiences a reduction with an increase in *Ri*. The thermal buoyancy provides additional momentum for the fluid, the flow is accelerated, and more heat is carried away at this specific surface point. Therefore, the thermal boundary layer should attenuate around the surface. For a large *Pr*, this decreasing trend of δ_T_ with *Ri* becomes insignificant. Particularly for a large *Pr*, for example *Pr* = 100, a negligible variation in δ_T_ with increasing *Ri* is observed, which indicates that the effect of thermal buoyancy on the formation of the thermal boundary layer can be neglected. For all *Ri*, δ_T_ shows a significant reduction with increasing *Pr*. These observations are similar to and also confirm those of the variation of isotherms with *Pr* presented in Figure 8 and Figure 9. With an increase in *Pr*, heat convection is more significant compared to heat conduction. More heat is effectively transferred downstream from the porous cylinder by convection. This eventually results in a reduction in the thermal boundary layer thickness.

The comparisons of the variation of δ_T_ with *D*a at different *Re* for *Pr* = 10 and *Ri* = 1 are depicted in Figure 10b. For all *R*e studied here, δ_T_ gradually decreases when *Da* increases. For a less permeable cylinder at a fixed *Re*, less fluid passes through the cylinder, and only a small part of heat can be transferred downstream. As a result, much heat is stored inside the cylinder, and the thermal boundary layer thickens around the cylinder surface. When the cylinder becomes more permeable, the resistance of the porous cylinder to the fluid becomes less significant. Correspondingly, the fluid velocity is accelerated, and the velocity gradient decreases, which leads to a reduction in the entropy production due to the viscous effect [50]. This accelerated fluid results in a large amount of convective heat transfer in this system, which ultimately narrows the temperature contours in the lateral sides (see Figure 8 and Figure 9) and thins the thermal boundary layer along the surface. For all *Da*, δ_T_ also shows a descending tendency when *Re* increases. For constant *Pr*, *Ri*, and *Da* and lower *Re*, the fluid velocity is relatively smaller, and heat transfer is mainly dominated by conduction. When *Re* becomes larger, fluid movement becomes pronounced, owing to the relatively larger inertial force, and convection is dominant in this situation. Correspondingly, the thermal boundary layer decreases, which also indicates a growth in entropy production owing to the increasing temperature gradient.

### 4.3. Heat Transfer Rate

#### 4.3.1. Local Nusselt Number

**Figure 11:**
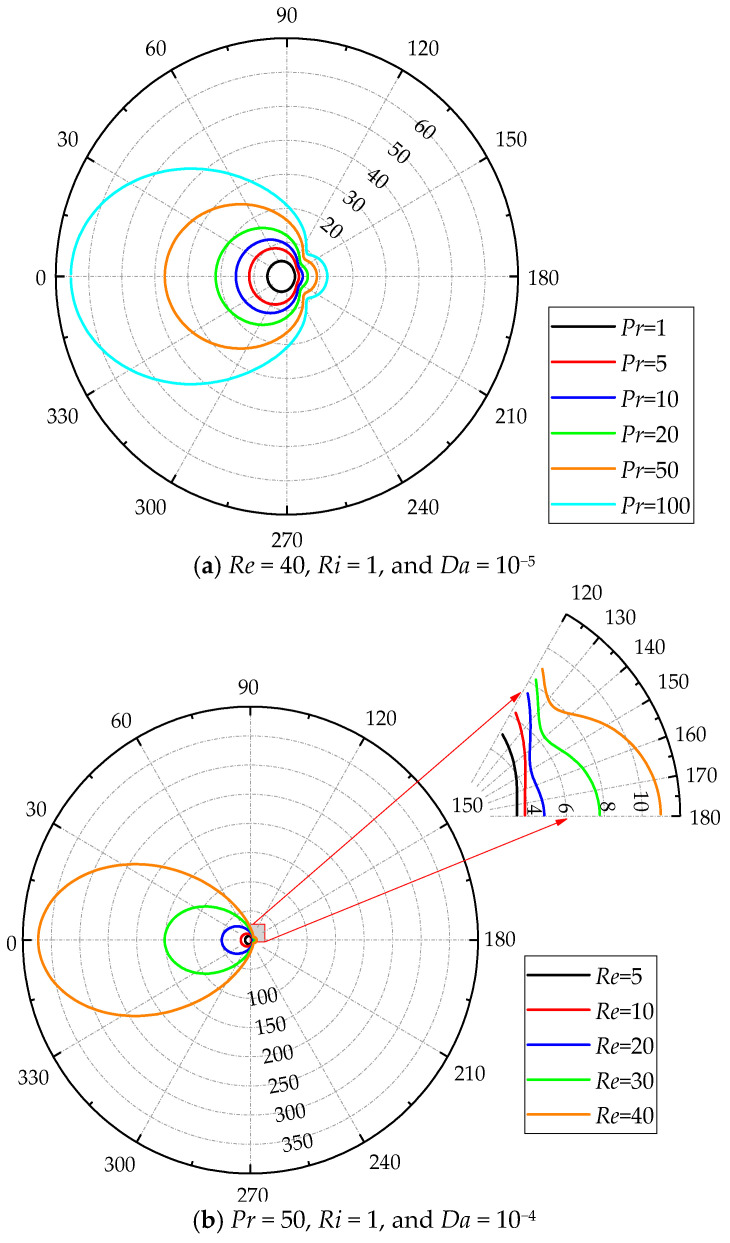

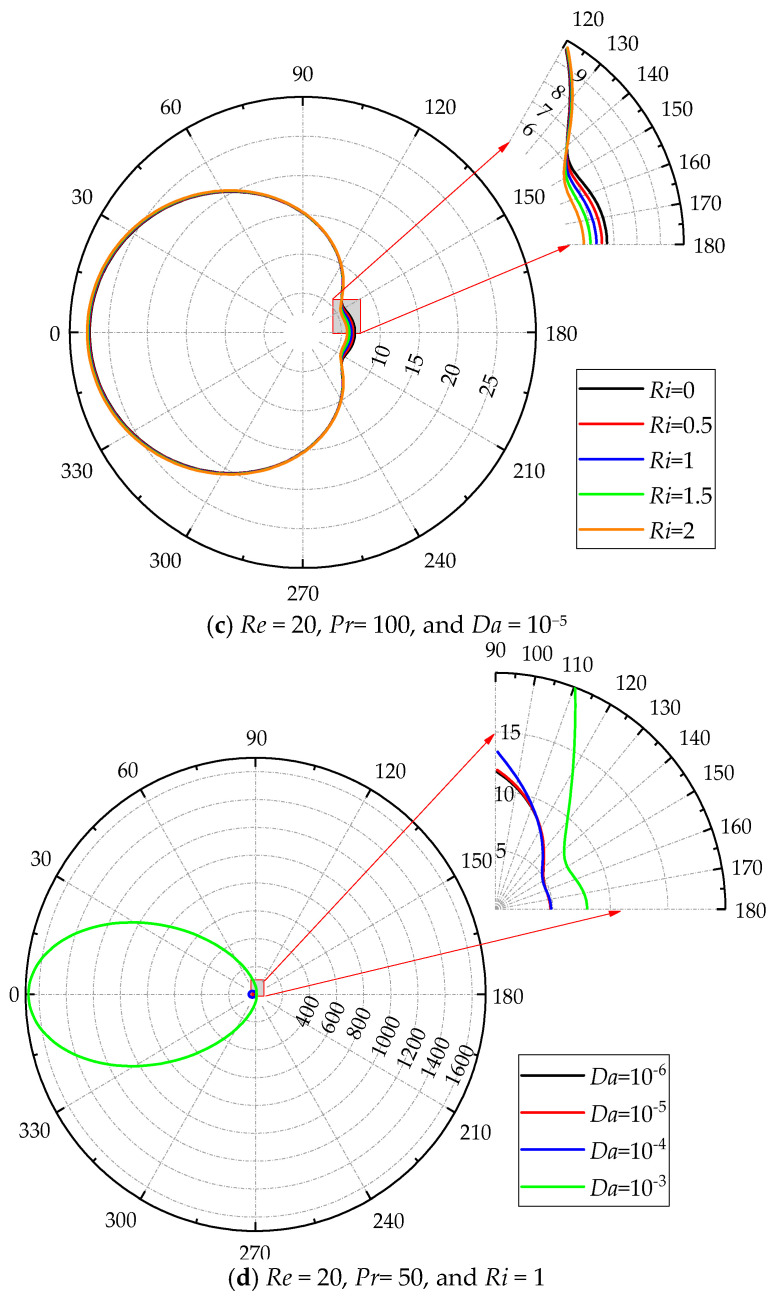
Variation of the local Nusselt number (the *Nu* scale is indicated on each circle with a dashed line, while the angular scale is provided outside of the circle with a solid line).

The Nusselt number (*Nu*) is a non-dimensional parameter to characterize the heat transfer rate in a thermal system, which is defined as

(10) $$Nu=\frac{hd}{k_{f}}=\frac{1}{4T|{}_{interface}},$$

where *h* is the heat transfer coefficient. The local heat transfer performance quantified by the local Nusselt number (*Nu*) along the porous cylinder surface is shown in Figure 11. To better present the distribution of *Nu* along the cylinder surface, the polar coordinate is chosen. The numbers distributed in the radial direction represents the magnitude of *Nu.*

Figure 11a shows the variations of *Nu* for different *Pr* at *Re* = 40, *Ri* = 1, and *Da* = 10^−5^. Large *Nu* is certainly noticed at the front of the cylinder for all *Pr*. For a small *Pr*, *Nu* gradually decreases along the cylinder surface from the front to the rear. The minimum *Nu* is found at the rear point. However, there is a jump in the distribution of *Nu* along the cylinder surface for a large *Pr*, i.e., *Nu* initially decreases and then increases from the front to the rear. The minimum *Nu* is just observed at the location where the jump occurs. When *Pr* increases, this phenomenon is significant. The positions of the occurrence of the jump in the distribution of *Nu* are in accordance with those of the onset of swells of the isotherms shown in Figure 8. Across this position, the crowded isotherms distribute near the cylinder, resulting in an increase in the temperature gradient. Therefore, *Nu* increases after the jump location. Moreover, the jump location is found to move upstream along the cylinder surface when *Pr* increases, which indicates that the positions of the onset of swells of the isotherms are greatly dependent on *Pr* in this situation. A similar observation is also identified for different *Re* shown in Figure 11b.

The variation of *Nu* with *Ri* is illustrated for a large *Pr* = 100 and constant *Re* = 20 and *Da* = 10^−5^ in Figure 11c. Different from the case of small *Pr* (see reference [18]), there is a turning point in the distribution of *Nu* along the cylinder surface, across which the trend of variation of *Nu* with increasing *Ri* becomes opposite. The concave isotherms behind the cylinder are relaxed and smoothed when *Ri* increases, which causes an increase in the distance between two neighboring isotherms behind the cylinder (see Figure 8 and Figure 9). As a result, the temperature gradient decreases. Therefore, *Nu* decreases with *Ri* after the turning point. The effect of *Da* on *Nu* along the cylinder surface at a larger *Pr* = 50 is shown in Figure 11d. *Nu* increases with *Da* at the front part of the cylinder. However, the distributions of the isotherms at the rear varying with *Da* are more complicated owing to the concave structure of the isotherms at larger *Pr*.

#### 4.3.2. Enhancement Ratio

The average Nusselt number (*Nu_ave_*) is defined as

(11) $$Nu_{ave}=\frac{1}{A}\int_{A}NudA,$$

where *A* is the surface area.

**Figure 12:**
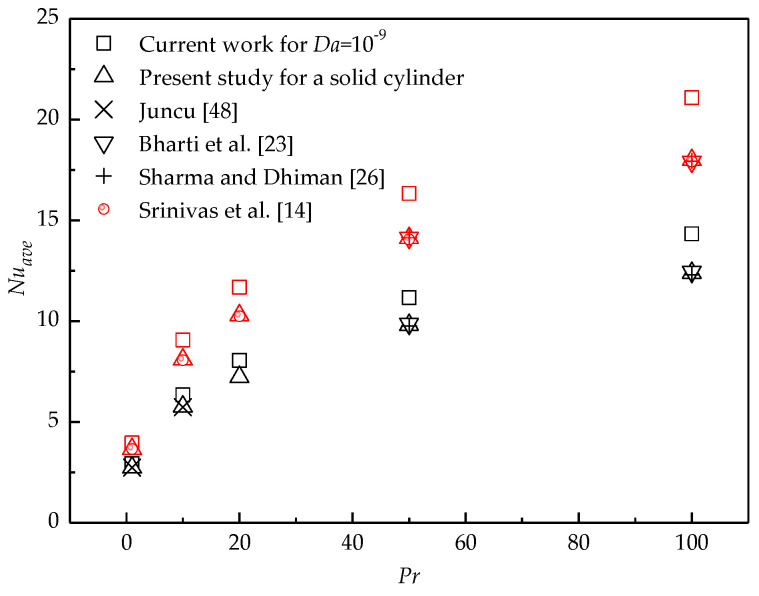
Comparisons of the present results with previous data for a heated solid cylinder (black scatters for *Re* = 20 and red scatters for *Re* = 40).

When *Da* is rather small, the cylinder almost becomes impermeable. To investigate the heat transfer difference between the present work for a porous cylinder with internal heat generation and the previous works for a solid cylinder with a constant surface temperature, the case of *Da* = 10^−9^ is performed for different *Pr* and *Re*. Simulations for flow around a solid cylinder with constant surface temperature are also carried out by the present method. The comparisons of the current results with the previous results for different *Pr* at *Re* = 20 (black scatters) and 40 (red scatters) in the forced convection regime (*Ri* = 0) are shown in Figure 12. It is seen that for a fixed *Re*, *Nu_ave_* for *Da* = 10^−9^ in the present study is larger than that of the previous works for all *Pr* studied here. When *Da* increases, more fluid can penetrate the porous cylinder, which converts more heat downstream. Therefore, *Nu_ave_* for larger *Da* is much higher than that for the solid cylinder.

**Figure 13:**
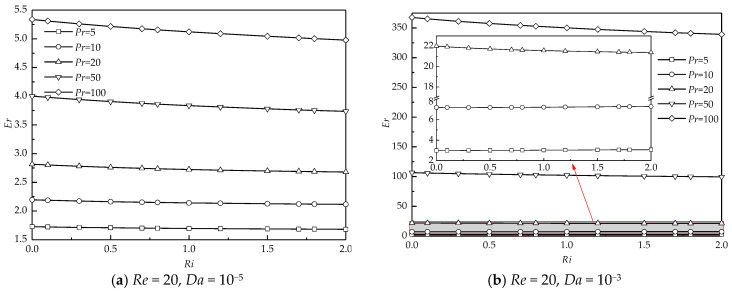

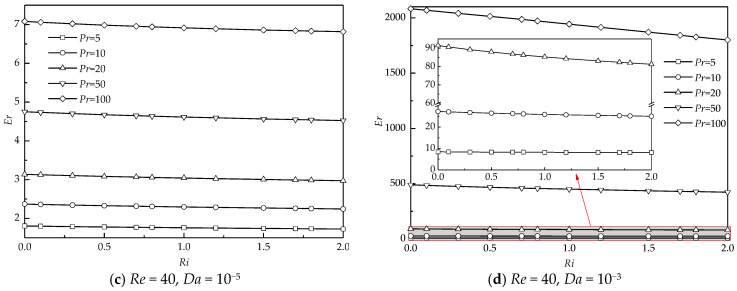
The enhancement ratio as a function of *Ri* at different *Pr*, *Re*, and *Da*.

To better depict the enhancement of heat transfer, the ratio of the average Nusselt number at various *Pr* (*Nu_ave_*) to that at *Pr* = 1 (Nu^*^_ave_) is defined as the enhancement ratio (*Er*) = *Nu_ave_*/Nu^*^_ave_. The variation of *Er* with *Ri* at different *Pr*, *Re*, and *Da* is illustrated in Figure 13. *Er* almost varies monotonously and linearly with increasing *Ri* for all *Pr*, *Re*, and *Da*. Figure 13a,b present the results at a smaller *Re* = 20. For a smaller *Da* =10^−5^ shown in Figure 13a, the variation of *Er* with *Ri* is insignificant for a small *Pr*. For a large *Pr*, the decreasing tendency of *Er* with *Ri* becomes significant, which means that changes in the strength of the thermal buoyancy at a large *Pr* have a relatively significant effect on *Er* compared to those at a small *Pr*. For all *Ri*, *Er* shows an increasing trend with *Pr*. An increase in *Pr* certainly decreases the thickness of the thermal boundary layer around the cylinder presented in Figure 10a, which significantly enhances the heat transfer rate. Similar results of the variation of *Er* with *Ri* at different *Pr* are also obtained at a larger *Da* = 10^−3^ (see Figure 13b).

Figure 13c,d show the dependence of *Er* on *Ri* and *Pr* at a larger *Re* = 40. For a less permeable case of *Da* =10^−5^ shown in Figure 13c, at a fixed *Ri* and *Pr*, the magnitude of *Er* increases compared to that at *Re* = 20, owing to the decreasing thickness of the thermal boundary layer at a larger *Re* shown in Figure 10b. When the cylinder becomes more permeable (see Figure 13d), *Er* significantly increases compared to that for *Da* =10^−5^ at constant *Ri* and *Pr*. However, the decreasing trend of *Er* with *Ri* is obviously witnessed, especially at a large *Pr*.

## 5. Conclusions

The present study numerically reveals the effect of the Prandtl number on the flow and heat transfer through and around a porous cylinder with internal heat generation in the mixed convection regime. The wake structure in terms of the streamlines, the wake length, the penetration depth, the isotherms, the boundary layer thickness, and the heat transfer rate characterized by the Nusselt number and enhancement ratio are mainly investigated in detail.

The numerical results show that a pair of the detached recirculating wakes occurs at an intermediate *Da* for a large *Ri* and a small *Pr* owing to the large base bleed. For a small *Ri* and a large *Pr*, the recirculating wake partially penetrates the porous cylinder. At a fixed *Re*, *Pr* is found to precipitate the growth of the recirculating wake. An increase in *Pr* weakens the effect of thermal buoyancy, which results in a reduction in the velocity through the porous cylinder and directly decreases the effect of base bleed. Less bleeding from the rear is not enough to support the entrainment need of the shear layer. Therefore, the large recirculating wake occurs.

The thermal field is significantly sensitive to *Pr*. For a small *Pr*, smooth isotherms sparely and widely enwrap the porous cylinder due to the relative dominance of thermal conduction. When *Pr* increases, the isotherms are no longer obedient, and they become concave in shape behind the cylinder. For a large *Pr*, these concave isotherms are significantly highlighted. Similar results are also observed with an increase in *Re*. The positions of the occurrence of swells of the isotherms almost match the streamlines where the flow separation occurs. Moreover, the present results suggest that the isotherms with lower temperature initially become concave for a larger *Da*. Thermal buoyancy provides the additional momentum to this system and tends to appease and relax the concave structure of the isotherms.

The local heat transfer rate is witnessed to minimize at the position where swells of the isotherms form. After this position, dense isotherms distribute near the cylinder, which results in a relatively large temperature gradient. Therefore, the local heat transfer rate increases across this position. An increase in *Pr* means that there is a much thinner thermal boundary layer relative to the momentum boundary layer. The thin thermal boundary layer results in a large temperature gradient. Thus, the heat transfer rate enhances.

## Figures and Tables

**Table 1:** Grid-independence analysis for thermal flow through and around a porous cylinder. *Nu_ave_*: average Nusselt number.

Case	Total Size	Grid Size					*Nu_ave_*
		Block1	Block2	Block3	Block4	Block5	
Case 1	15,080	80 × 60	80 × 55	80 × 40	80 × 15	20 × 20	4.36899
Case 2	48,420	160 × 140	160 × 70	160 × 50	160 × 20	40 × 40	4.35218 (0.7004%)
Case 3	100,160	240 × 220	240 × 80	240 × 60	240 × 30	60 × 60	4.34866 (0.3520%)
Case 4	170,300	320 × 300	320 × 90	320 × 70	320 × 40	80 × 80	4.35157 (0.4676%)

## Abbreviations

Nomenclature	
*g*	gravitational acceleration, [m/s^2^]
*q*′″	heat source, [W/m^3^]
*d*	diameter of cylinder, [m]
*A*	surface area, [m^2^]
*K*	permeability of cylinder, [m^2^]
*k_f_*	thermal conductivity of fluid, [W/(m·°C)]
*k_e_*	effective thermal conductivity, [W/(m·°C)]
*R_c_*	thermal conductivity ratio
*h*	heat transfer coefficient, [W/(m^2^·°C)]
*x*	dimensional horizontal coordinate, [m]
*y*	dimensional vertical coordinate, [m]
*u*	dimensional *x*-component velocity, [m/s]
v	dimensional *y*-component velocity, [m/s]
*p*	pressure, [Pa]
*T**	temperature, [°C]
*X*	dimensionless horizontal coordinate
*Y*	dimensionless vertical coordinate
*U*	dimensionless x-component velocity
*V*	dimensionless y-component velocity
*P*	dimensionless pressure
*T*	dimensionless temperature
*Lw*	the wake length
*Lp*	the penetration depth
*Re*	Reynolds number
*Pr*	Prandtl number
*Gr*	Grashof number
*Ri*	Richardson number
*Da*	Darcy number
*Nu*	Nusselt number
*Er*	enhancement ratio
*n*	the dimensionless normal direction
Greek symbols	
*α*	thermal diffusivity, [m^2^/s]
*β*	thermal expansion coefficient, [°C^−1^]
υ	fluid kinematic viscosity, [m^2^/s]
*ε*	fluid kinematic viscosity porosity
*ρ*	fluid density, [kg/m^3^]
δ	the thickness of the thermal boundary layer
ϕ	a binary constant
Subscripts	
∞	free stream
*ave*	average
